# The Impact of Crohn’s Perianal Fistula on Quality of Life: Results of an International Patient Survey

**DOI:** 10.1093/crocol/otad036

**Published:** 2023-07-25

**Authors:** Antonino Spinelli, Henit Yanai, Paolo Girardi, Slobodan Milicevic, Michele Carvello, Annalisa Maroli, Luisa Avedano

**Affiliations:** Department of Biomedical Sciences, Humanitas University, Via Rita Levi Montalcini 4, 20090 Pieve Emanuele, Milan, Italy; Division of Colon and Rectal Surgery, IRCCS Humanitas Research Hospital, Via Manzoni 56, 20089 Rozzano, Milan, Italy; IBD Center, Division of Gastroenterology, Rabin Medical Center, 4941492 Petah Tikva, Israel; Sackler Faculty of Medicine, Tel Aviv University, 6997801 Tel Aviv, Israel; Department of Environmental Science, Informatics and Statistics, Ca’ Foscari University of Venice, 30123 Venice, Italy; Takeda Pharmaceuticals International AG, Glattpark-Opfikon, 8152 Zürich, Switzerland; Department of Biomedical Sciences, Humanitas University, Via Rita Levi Montalcini 4, 20090 Pieve Emanuele, Milan, Italy; Division of Colon and Rectal Surgery, IRCCS Humanitas Research Hospital, Via Manzoni 56, 20089 Rozzano, Milan, Italy; Division of Colon and Rectal Surgery, IRCCS Humanitas Research Hospital, Via Manzoni 56, 20089 Rozzano, Milan, Italy; European Federation of Crohn’s & Ulcerative Colitis Associations, 1000 Brussels, Belgium

**Keywords:** Crohn’s disease, psychological distress, relationships, patient-reported outcomes research

## Abstract

**Background:**

Crohn’s perianal fistula is a disabling manifestation of Crohn’s disease. However, the additional burden of perianal fistula on patients with only Crohn’s disease remains to be addressed. This patient-reported survey considered outcomes of two domains: “diagnosis” (eg, symptoms) and “living with the disease” (eg, quality of life, well-being, and relationships).

**Methods:**

Patients with perianal fistula and Crohn’s disease completed an online, self-selective, anonymous, 46-item survey available in 11 languages hosted on the European Federation of Crohn’s & Ulcerative Colitis Associations and national patient association websites. The survey was conducted between July and December 2019 in Europe and other regions. Likert scales and closed questions were used to assess outcomes.

**Results:**

Of the 820 respondents with Crohn’s disease (67.2% women; median age, 40.0 years), 532 (64.9%) reported the presence of perianal fistula. Patients with perianal fistula reported a greater impact on overall quality of life (*P* < .001), well-being (*P* < .001), relationships (*P* < .001), social life (*P* = .001), and work life (*P* = .012) than patients with only Crohn’s disease.

**Conclusions:**

Perianal fistulas impact several domains of the life of patients with Crohn’s disease. These results may help healthcare practitioners plan therapeutic strategies that address the symptomatic and psychological burden experienced by patients with perianal fistulizing Crohn’s disease.

## Introduction

Crohn’s disease (CD) is a chronic inflammatory disease of the gastrointestinal tract characterized by transmural ulceration that can disrupt the mucosal integrity of the intestine and anal canal,^1^ leading to complications such as abscesses and fistulas.^2^ Crohn’s perianal fistula (CPF) is a debilitating complication of CD.^3^ Approximately 35.0% of patients with CD have ≥1 fistula,^4^ and an estimated 70.0%–80.0% of perianal fistulas are complex.^5^ CPF can cause symptoms such as fecal incontinence, rectal pain, swelling, and fever, which may significantly affect a patient’s social performance, sexual function, and normal life activities.^5,6^ Furthermore, complex fistulas involve the upper part of the sphincters, have multiple external openings with pain and fluctuations suggestive of a perianal abscess, and may be associated with a rectovaginal fistula or an anorectal stricture.^5,7^ From the Goligher classification in 1875 to the recent American College of Gastroenterology classification,^8^ there are several classifications of CPF. Currently, there is no consensus on the classification system that should be used to establish a clinically relevant categorization of CPF to determine appropriate treatment strategies.^9^ Thus, treatment of CPF is challenging and complex, and patients are often refractory to conventional therapies such as immunomodulators, antibiotics, and biologics, including anti-tumor necrosis factor agents.^5,7,8^

Notably, patients with CPF have a high disease burden due to the impact on the health-related quality of life (QoL) and increased healthcare resource use and associated costs.^5,10,11^ In recent years, patient-centered and shared decision-making (SDM) approaches have become more prominent treatment paradigms, particularly for complex diseases.^12–14^ Improved healthcare practitioner (HCP) awareness and understanding of patients’ experience of disease burden may provide guidance to tailor an individualized treatment approach and allow for patients to receive personalized interventions and care in a multidisciplinary context that extends beyond pharmacological treatment. However, to date, the experiential burden that CPF exerts on patients relative to those with CD only remains to be fully elucidated. Indeed, few studies have addressed patients’ perspectives on the impact of CPF on overall QoL, and there is a paucity of patient-reported outcome measures specifically designed for patients with CPF.^11,15,16^ To address this prevailing unmet need, the European Federation of Crohn’s & Ulcerative Colitis Associations (EFCCA) developed and conducted an international, patient-reported survey to assess the additional burden and impact of CPF in patients with CD.

## Materials and Methods

### Study Design

The study conforms to the ethical guidelines of the 1975 Declaration of Helsinki (6th revision, 2008). Informed consent was implied from each patient included in the study through their explicit agreement to take part in the survey.

An anonymous, self-selective, 46-item survey was developed by EFCCA in collaboration with medical and patient representatives (Supplementary Data). Survey methodology and results have been previously presented.^17,18^ The survey was hosted on the EFCCA website and national inflammatory bowel disease (IBD) patient association websites from July 15 through December 31, 2019, and was published in the following languages: English, French, German, Greek, Hebrew, Italian, Polish, Portuguese, Romanian, Spanish, and Slovenian.

### Patients

To assess the additional burden of CPF relative to CD only, the study included patients with CD with or without CPF aged ≥18 years. Patients with CD only were included as a comparator group to assess differences in patient-reported outcomes between the groups. Patients self-reported their CD/CPF diagnoses based on their understanding of the diagnosis received from a physician; diagnoses were not independently verified. Patients with noncomplex or complex CPF were included in the study and pooled for analysis; therefore, all references to CPF in the current study refer to complex or noncomplex CPF.

### International Patient Survey

The survey structure is presented in Figure S1. In brief, following a 6-item screening section, multiple aspects of patient experience were assessed across 2 domains: “diagnosis” and “living with the disease.” The “diagnosis” section contained 19 items, including history and activity of CD, diagnosis, and treatment. Specific items were included for patients with CPF, such as the number of fistulas experienced by the patient, whether the fistula was complex, and whether rectovaginal fistulas were present. The “living with the disease” section contained 21 items, including impact on overall QoL, subjective well-being (including depression and anxiety), relationships with family and partner, social life, and work life. Scoring systems across the survey domains included close-ended questions and 5- and 10-point Likert scales (Supplementary Data).

### Statistical Analysis

Statistical analysis was conducted after aggregating responses from all participating regions. Due to the sample imbalance across countries and regions and the lack of a concrete guideline to distinguish noncomplex and complex CPF,^9^ the analysis was performed in an aggregate format, with pooling of patients reporting noncomplex and complex CPF. Data with nonnormal distributions (eg, Likert scale outcomes) were summarized using median and interquartile range (IQR: first quartile, third quartile). Differences in continuous variables between the CPF and CD-only groups and between gender were assessed using the Mann–Whitney *U*-test. For categorical variables, the Chi-squared test was used to test for significant differences in frequencies. A mixed-effects cumulative logistic regression model was used to assess the factors statistically associated with subjective well-being scores. The final model included gender, age, presence of perianal fistulas, current CD situation, CD duration, CD information, and type of work as fixed effects, while, to take into account the sampling strategy, the country of origin was included in the model as a random effect. For model selection, the Akaike Information Criterion was used with a backward-selection procedure. This is a statistical method for evaluating how well a model fits the data it was generated from and therefore measures the relative quality of the statistical model. Odds ratios were calculated by exponentiating the estimated coefficients with a relative confidence interval of 95%. The level of statistical significance for the tests and regression model was set at 5%. All analyses were performed using R, version 4.0.

## Results

### Demographic and Clinical Characteristics

Of the 820 respondents with CD, 288 (35.1%) reported CD only and 532 (64.9%) reported CPF. Overall, 67.2% of respondents were women, and the median age was 40.0 years. One participant who stated “Can’t remember” for the complexity of CPF was excluded from the analysis due to missing values for the variables. Most respondents were from Italy (*n* = 162, 19.8%) and Spain (*n* = 160, 19.5%), and a higher proportion of respondents from Italy and Spain reported CPF than any other participating country (Table 1). More patients with CPF reported active CD than those with CD only (Table 1). A disease duration of >15 years was reported by 41.7% and 33.7% of patients with CPF and CD only, respectively (Table 1). Gastroenterologists were the HCPs most often involved in the diagnosis and treatment of CD and CPF. Respondents with CPF and CD only reported that they were equally well informed of their health condition (Table 1). Demographic and clinical characteristics stratified by gender are reported in Table S1.

**Table 1.** Demographics, disease characteristics, diagnosis, and treatment patterns.

	Overall (*n* = 820)	Patients with CPF (*n* = 532)	Patients with CD only(*n* = 288)
Country or residence, *n* (%)			
Italy	162 (19.8)	133 (25.0)	29 (10.1)
Spain	160 (19.5)	122 (22.9)	38 (13.2)
Portugal	93 (11.3)	55 (10.3)	38 (13.2)
Greece	85 (10.4)	52 (9.8)	33 (11.5)
Poland	70 (8.5)	40 (7.5)	30 (10.4)
Slovenia	61 (7.4)	30 (5.6)	31 (10.8)
France	59 (7.2)	17 (3.2)	42 (14.6)
Romania	37 (4.5)	16 (3.0)	21 (7.3)
Belgium	19 (2.3)	8 (1.5)	11 (3.8)
Austria	14 (1.7)	9 (1.7)	5 (1.7)
Other countries	60 (7.3)	50 (9.4)	10 (3.5)
Gender, *n* (%)			
Women	551 (67.2)	352 (66.2)	199 (69.1)
Men	269 (32.8)	180 (33.8)	89 (30.9)
Age, years, median (IQR)	40.0 (32.0, 49.0)	40.0 (32.0, 49.0)	40.0 (32.0, 48.0)
Current CD situation, *n* (%)			
Not active	492 (60.0)	307 (57.7)	185 (64.2)
Active	311 (37.9)	217 (40.8)	94 (32.6)
Don’t know	17 (2.1)	8 (1.5)	9 (3.1)
CD duration, years, *n* (%)			
<1	66 (8.0)	31 (5.8)	35 (12.2)
1–5	177 (21.6)	114 (21.4)	63 (21.9)
6–10	148 (18.0)	88 (16.5)	60 (20.8)
11–15	110 (13.4)	77 (14.5)	33 (11.5)
>15	319 (38.9)	222 (41.7)	97 (33.7)
Diagnosis by specialty, *n* (%)			
Gastroenterologist	648 (79.0)	411 (77.3)	237 (82.3)
Family doctor	22 (2.7)	13 (2.4)	9 (3.1)
Surgeon	90 (11.0)	68 (12.8)	22 (7.6)
Other physicians	60 (7.3)	40 (7.5)	20 (6.9)
Current treatment specialty, *n* (%)			
Family doctor	93 (11.3)	62 (11.7)	31 (10.8)
Gastroenterologist	752 (91.7)	482 (90.6)	270 (93.8)
Surgeon	202 (24.6)	191 (35.9)	11 (3.8)
Other physicians	71 (8.7)	56 (10.5)	15 (5.2)
Level of importance doctor places on treatment, *n* (%)			
Very high importance	343 (41.8)	220 (41.4)	123 (42.7)
Quite high importance	264 (32.2)	168 (31.6)	96 (33.3)
Some importance	136 (16.6)	96 (18.0)	40 (13.9)
Limited importance	53 (6.5)	33 (6.2)	20 (6.9)
No importance	19 (2.3)	11 (2.1)	8 (2.8)
Don’t know	5 (0.6)	4 (0.8)	1 (0.3)
How well informed are you about CD?^a^Median (IQR)	8.00 (7.00, 9.00)	8.00 (7.00, 9.00)	8.00 (7.00, 9.00)

Abbreviations: CD, Crohn’s disease; CPF, Crohn’s perianal fistula; IQR, interquartile range.

All variables include self-reported information. Other countries include Colombia, the Dominican Republic, Greenland, Guadeloupe, Israel, Mexico, and the United States of America.

^a^Based on a Likert scale of 1–10; 1 indicating “not informed at all” and 10, “extremely well informed.”

### Survey Section A: Diagnosis

**Figure 1.**
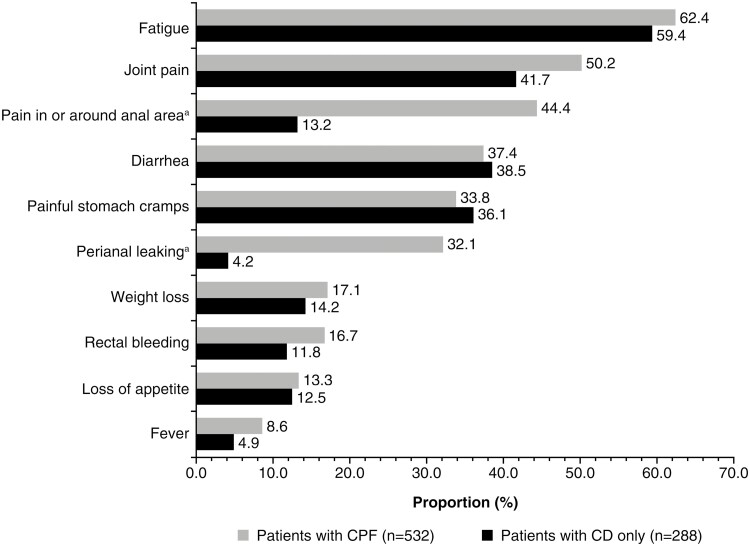
Current symptoms among patients with Crohn’s perianal fistula versus Crohn’s disease only. ^a^*P* < .001, derived using Pearson’s Chi-squared test. CD, Crohn’s disease; CPF, Crohn’s perianal fistula.

Of the patients with CPF, 272 (51.1%) reported complex fistulas. Fatigue was the most common symptom in patients with CD only and CPF, followed by joint pain (Figure 1). As expected, a higher proportion of patients with CPF experienced pain in or around the anal area and leakage or drainage in the perianal area than those with CD only (Figure 1). When CPF symptoms were stratified by gender, more women reported fatigue, joint pain, diarrhea, and painful stomach cramps than men (Table S2).

### Survey Section B: Living With the Disease

#### Overall QoL and subjective well-being

**Figure 2.**
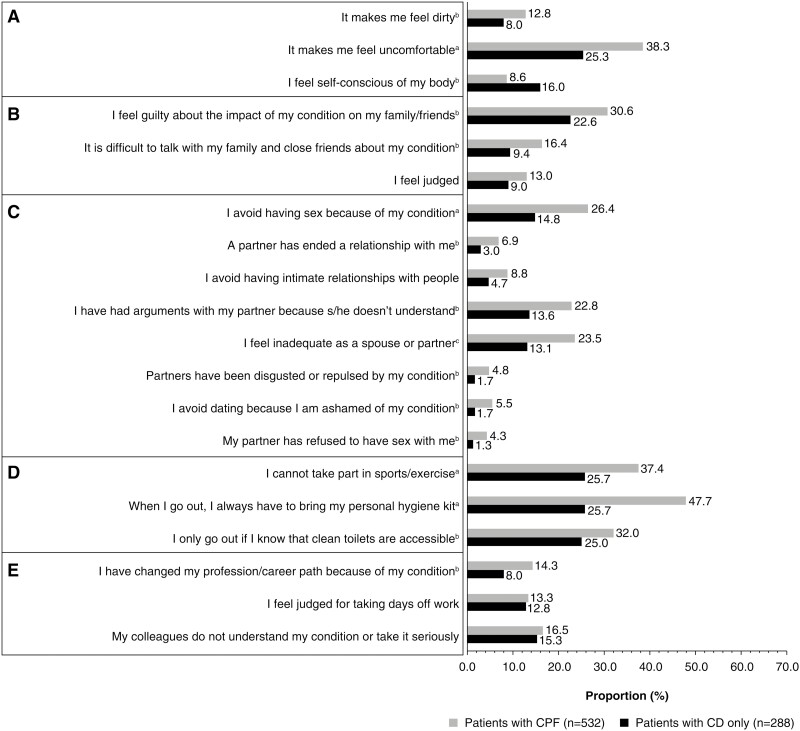
Impact of Crohn’s perianal fistula versus Crohn’s disease only. (A) Subjective well-being. (B) Relationship with family/friends. (C) Relationship with partner (number of respondents for relationship with partner is based on presence [*n* = 421] or absence [*n* = 236] of partner and presence of fistula). (D) Social life. (E) Work life. ^a^*P* < .05, ^b^*P* < .001, ^c^*P* = .001 derived using Mann–Whitney *U*-test, Pearson’s Chi-squared test, and Fisher’s exact test. CD, Crohn’s disease; CPF, Crohn’s perianal fistula.

Patients with CPF reported a higher impact on overall QoL than patients with CD only (CD only: median [IQR]: 6 [4–8]; CPF median [IQR]: 7 [5–9]; *P* < .001) and reported a greater impact on subjective well-being (*P* < .001; Table 2). More patients with CPF than CD only reported feeling “dirty” and “uncomfortable” (Figure 2). Among patients with CPF, there was no difference in subjective well-being between genders (Table S3). More women with CPF reported feeling helpless and less understood by people than men (Figure S2). In the logistic regression model, CPF and active CD were associated with an impact on subjective well-being. Additionally, specific categories of employment (retired, unemployed, self-employed, or undefined) were associated with an impact on subjective well-being versus full-time employment (Table 3).

**Table 2.** Impact scores for survey outcomes in CPF versus CD only groups.

Survey outcome, median (IQR)	*N*	Overall	Patients with CPF (*n* = 532)	Patients with CD only (*n* = 288)	*P*-value
Subjective well-being	820	7.00 (5.00, 9.00)	7.00 (5.00, 9.00)	6.00 (4.00, 8.00)	<.001
Relationships with family/friends	820	5.00 (3.00, 8.00)	6.00 (3.00, 8.00)	5.00 (2.00, 7.00)	<.001
Relationship with partner^a^	657	5.00 (2.00, 7.00)	5.00 (2.00, 8.00)	3.00 (1.00, 6.00)	<.001
Social life	820	5.00 (3.00, 8.00)	6.00 (3.00, 8.00)	5.00 (2.75, 7.00)	.001
Working life^b^	566	6.00 (3.00, 8.00)	6.00 (3.00, 8.00)	5.00 (3.00, 7.00)	.012

Abbreviations: CD, Crohn’s disease; CPF, Crohn’s perianal fistula; IQR, interquartile range.

Based on a Likert scale of 1–10; 1 indicating “no impact at all” and 10, “very big impact.”

^a^ *n* = 163 respondents reported not being in a relationship with a partner.

^b^ *n* = 254 respondents were not included in the work-life analysis, which included respondents who were unemployed [*n* = 88], retired [*n* = 83], homemakers/housewives [*n* = 35], or had “*other*” employment status.

**Table 3.** Estimated ORs for factors influencing subjective well-being.

Predictors	OR	95% CI	*P-*value
Gender (Female [reference])	1.00	—	**—**
Gender (Male)	0.62	0.47–0.81	<.001^a^
Only Crohn’s disease (reference)	1.00	—	**—**
Crohn disease with CPF (Yes)	1.52	1.16–1.97	.002^a^
CD situation (Not active)	1.00	—	—
CD situation (Active)	3.80	2.91–4.96	<.001 ^a^
CD situation (Don’t know]	2.62	0.96–7.19	.061
Type of employment (Full-time employed [reference])	1.00	—	—
Type of employment (Home maker/housewife)	1.62	0.85–3.09	.139
Type of employment (Other)	2.25	1.27–3.96	.005^a^
Type of employment (Part-time employed)	1.26	0.83–1.92	.282
Type of employment (Retired)	1.71	1.12–2.62	.013^a^
Type of employment (Self-employed)	1.83	1.08–3.11	.025^a^
Type of employment (Student)	1.25	0.74–2.11	.404
Type of employment (Unemployed)	1.90	1.24–2.89	.003 ^a^
Random effects			
*σ* ^2^	3.29		
τ_00country_	0.05		
Intra-class correlation	0.01		
*N*_country_	11		
Observations	820		
Marginal *R*^2^/Conditional *R*^2^	0.168/0.179		

Abbreviations: CD, Crohn’s disease; CI, confidence interval; CPF, Crohn’s perianal fistula; OR, odds ratio.

^a^Indicates statistical significance.

#### Relationships

Patients with CPF reported a greater impact on relationships with family and friends than those with CD only (Table 2). More patients with CPF reported feeling guilty about the impact of the condition on family and friends and found it more difficult to talk with family and friends about their condition than those with CD only (Figure 2). Women reported a greater impact of CPF on relationships with family and friends than men (Table S3). More women with CPF reported needing support for everyday tasks and that they “talk a lot with their family and close friends about their condition, which helps them a lot” more often than men. More women than men reported that their family and close friends did not understand their situation (Figure S2).

A total of 236 (81.9%) patients with CD only and 421 with CPF (79.1%) reported having a partner. Patients with CPF reported a greater impact on their relationships with their partners than patients with CD only (Table 2), with higher impact scores on all items (Figure 2). Women reported a greater impact of CPF on relationships with their partner than men (Table S3). More women than men reported that they avoided having sexual intercourse with their partner but indicated that the condition strengthened their relationship with their partner (Figure S2).

#### Social and work life

Patients with CPF reported a greater impact on social life than those with CD only (Table 2). Compared with patients with CD only, more patients with CPF reported that they were unable to take part in sports and exercise, always had to carry a personal hygiene kit when leaving their home, and that they only left their home if they knew clean toilets were accessible (Figure 2). Women reported a greater impact of CPF on their social lives than men (Table S3). More women than men reported carrying a personal hygiene kit when leaving their homes (Figure S2).

A total of 566 respondents were employed (full time, *n* = 381 [46.5%]; part time, *n* = 86 [10.5%]; self-employed, *n* = 51 [6.2%], and students, *n* = 48 [5.9%]) (Table 4). Respondents who were unemployed or retired were excluded from the work-life analysis. Additionally, respondents who were homemakers or with other employment status were also excluded from the work analysis since information on their work was not collected during the survey. Patients with CPF reported a greater impact on work life than those with CD only (Table 2, Figure 2). More patients with CPF reported having changed their profession/career due to their condition than those with CD only (Figure 2). Patients with CPF also reported a higher number of days off work due to their condition than those with CD only (Table 4). Women reported a greater impact on work life than men (Table S3).

**Table 4.** Employment status and work-life challenges of patients with CPF versus CD only.

	Overall (*n* = 820)	Patients with CPF (*n* = 532)	Patients with CD only (*n* = 288)	*P*-value
Type of employment, *n* (%)				
Full-time employed	381 (46.5)	243 (45.7)	138 (47.9)	
Homemaker/housewife	35 (4.3)	21 (3.9)	14 (4.9)	
Part-time employed	86 (10.5)	51 (9.6)	35 (12.2)	
Retired	83 (10.1)	59 (11.1)	24 (8.3)	
Self-employed	51 (6.2)	39 (7.3)	12 (4.2)	
Student	48 (5.9)	26 (4.9)	22 (7.6)	
Unemployed	88 (10.7)	60 (11.3)	28 (9.7)	
Other employment	48 (5.9)	33 (6.2)	15 (5.2)	

Median (IQR)	Overall (*n* = 566)	Patients with CPF (*n* = 359)	Patients with CD only(*n* = 207)	*P*-value
Because of my condition, the stresses of my job were much harder to handle	4.00 (2.00, 4.00)	4.00 (3.00, 5.00)	4.00 (2.00, 4.00)	.343
Despite having my condition, I was able to finish hard tasks in my work	4.00 (4.00, 5.00)	4.00 (3.50, 5.00)	4.00 (4.00, 5.00)	.677
My condition distracted me from taking pleasure in my work	3.00 (2.00, 4.00)	3.00 (2.00, 4.00)	3.00 (2.00, 4.00)	.051
I felt hopeless about finishing certain work tasks due to my condition	3.00 (2.00, 4.00)	3.00 (2.00, 4.00)	2.00 (2.00, 4.00)	.036
At work, I was able to focus on achieving my goals despite my condition	4.00 (3.00, 5.00)	4.00 (3.00, 5.00)	4.00 (3.00, 5.00)	.607
Despite having my condition, I felt energetic enough to complete all my work	3.00 (2.00, 4.00)	3.00 (2.00, 4.00)	4.00 (2.00, 4.00)	.585
Days off due to CPF	0.00 (0.00, 3.00)	0.00 (0.00, 8.00)	0.00 (0.00, 0.00)	<.001
Days off due to CD	3.00 (0.00, 12.00)	3.00 (0.00, 12.00)	3.00 (0.00, 10.00)	.196
Days off due to CD or CPF	5.00 (0.00, 20.00)	7.00 (0.00, 22.00)	3.00 (0.00, 11.00)	<.001

Abbreviations: CD, Crohn disease; CPF, Crohn’s perianal fistula; IQR, interquartile range.

Work experience was assessed using a 5-point scale (1, strongly disagree and 5, strongly agree) with a recall period of 1 month. The recall period for days off due to CD, CPF, or either was 6 months. *n* = 254 respondents were not included in the work-life analysis, which included respondents who were unemployed (*n* = 88), retired (*n* = 83), homemakers/housewives (*n* = 35), or had “*other*” employment status.

## Discussion

Broadly, it is now well recognized that CD is associated with a substantial burden on patients and healthcare systems.^19,20^ The presentation of perianal fistulas in CD can result in high morbidity and may infer additional burden.^10,21^ To elucidate the experiential aspects of CPF relative to CD only, the current study reports the results of a patient-reported survey that characterized the real-world impact (ie, patients’ health experience captured outside the clinical trial setting) of CPF across 2 broad domains, “diagnosis” and “living with the disease.” The study was conducted to address the acute unmet need for the development of patient-reported outcome measures in patients with CPF. Of note, most patients with CD experienced CPF, confirming that CPF is a pervasive complication of CD.^4,5^ Overall, the current study demonstrates significant differences between CD-only and CPF groups across multiple survey items, which indicates that CPF infers an increased psychosocial burden relative to CD only.

Fatigue is a well-established symptom of CD,^22,23^ and in the current survey, it was the most common symptom for both patients with CD only and CPF. Fatigue has been strongly associated with poor QoL among patients with CD independent of disease activity^24^ and is recognized as a patient-reported barrier toward the pursuit of hobbies and interests.^15^ Notably, common symptoms such as fatigue had a similar prevalence across both the CPF and CD-only groups. However, as anticipated, some common fistula symptoms such as perianal pain and leakage/drainage were reported by a significantly higher proportion of patients with CPF than those with CD only. In this present study, 1 in 3 respondents with CPF reported perianal leakage. In a recent qualitative study that aimed to understand the experience of patients living with CPF and its impact on QoL, patients reported that perianal leakage caused embarrassment and concerns due to its odor and visibility on the surrounding skin and clothing.^15^ Additionally, 1 in 2 respondents with CPF in our study reported perianal pain, which may be disease related or could be the result of side effects, recovery after invasive interventions for CPF, particularly following drainage or lay-open procedures, or seton placement.^3,15,25^

It is now well recognized that patients with CD experience psychological distress, including perceived stigmatization.^26–28^ However, evaluation of the psychological symptoms associated with CPF remains rare.^29^ By including items for QoL and subjective well-being, the current survey demonstrated that patients with CPF experience significant psychological distress compared with those with CD only. Patients with CPF had a poor self-concept and reported feeling more uncomfortable and dirtier than patients with CD only, while negative affect (eg, anxiety, depression, embarrassment) was common. Moreover, the heightened emotional dysfunction in CPF was supported by the results of the logistic regression model, wherein the presence of CPF was associated with an increased impact on subjective well-being relative to CD only. Of note, women indicated a greater impact on their subjective well-being than men, which supports previous literature reporting gender differences in QoL between women and men with IBD.^21^ Additionally, respondents from a qualitative survey indicated that they could not wear tight clothing owing to discomfort due to CPF, and women, in particular, reported feeling that their outfit choices were limited, further impacting their self-confidence and self-esteem.^15^

Our study showed that CPF has both positive and negative influences on relationships with family and friends. Respondents indicated that although they avoided making plans and missed family gatherings, they also felt that their family and close friends understood their condition. More pronounced negative consequences of CPF were also reported for relationships with partners, where the respondents indicated that they avoided sexual intercourse, had experienced a partner ending a relationship, and had arguments with their partner more often than those with CD only. Women reported a greater impact of CPF on relationships compared with men. Generally, while women seem to experience sexual dysfunction with limited improvement independent of disease activity over time,^30^ the literature also indicates a high level of anxiety around sexual intimacy, which is often related to the fear of leakage or unpleasant odor. Therefore, their emotional well-being is negatively impacted, leading to restricted sexual activities and desires.^15^

Regarding social life, patients with CPF reported a need for “toilet mapping” that restricted their participation in social events and corroborates previous findings regarding the behavioral consequences of living with IBD.^31,32^ Furthermore, CPF affected working life, resulting in respondents taking time off work, having restricted options in job roles, and having to change jobs or profession. This finding is supported by a population-based study in Canada that indicated a strong association between reduced productivity at work and lower QoL and emotional distress in patients with IBD.^33^

The current results have ramifications for clinical practice. Broadly, the survey illustrates that many patients have a good understanding of their disease and are, therefore, well placed to be involved in treatment choices during SDM.^12–14^ The areas of increased symptomatic burden in CPF highlighted in the current study (eg, perianal pain and leakage/drainage symptoms) may help HCPs further understand patients’ perspectives and develop accurate treatment strategies in conjunction with patients that include long-term goals such as CPF healing and improvement of QoL. Moreover, the diagnosis and management of CPF require a multidisciplinary approach, which involves cooperation between different specialized professionals participating in the treatment planning and adequate care of patients with CD/CPF. Nevertheless, despite the complexity and severity of the disease, most patients with CPF in the current study were diagnosed (77.3%) or treated (90.6%) by a gastroenterologist. However, it is important to note that these patients with CPF may not have been solely treated by gastroenterologists; instead, their treatment may have involved a collaborative effort between gastroenterologists and surgeons for interim surgical intervention such as surgical drainage, which is often required for effective management of this condition.^34,35^ Furthermore, as patient-reported outcome measures for CPF remain rare, the current survey has the potential to be applied clinically as a baseline measure of CD/CPF activity and patient-reported QoL. Subsequently, it could also be administered to guide treatment selection and monitor the effectiveness of therapeutic interventions.

The current study had several strengths. Recently, one of the first validated measures (the Crohn’s Anal Fistula Quality of Life [CAF-QoL] scale) to assess QoL in CPF has been published in English only using data from 211 patients in the United Kingdom.^11^ The development of the CAF-QoL represents an important step in addressing the prevailing unmet need. However, at the time of writing, the current study was the first to assess CPF-related QoL in a large and diverse patient sample from more than 33 countries and in a multitude of languages. The additional value of the current study is the inclusion of patients with CD only, which allowed for the experience of patients with CPF to be characterized against the baseline of CD only. Furthermore, the large number of patients who responded to the survey highlights that patients with CPF and/or CD clearly feel the need to communicate their disease burden; the illustration of this point is a strength of the current study.

The limitations of this study include self-reported CPF and CD diagnoses that were not independently verified by an HCP. Moreover, it was unclear whether patients with CD only had previously experienced perianal fistulas. However, despite patients reporting a high level of disease understanding, it is important to acknowledge that self-reporting of diagnoses may be subject to the possibility of recall bias. Furthermore, as we report the initial stages of survey development and administration, validation should be conducted in future studies to establish the psychometric properties of the survey (eg, test–retest reliability, content and construct validity). In addition, there was an imbalance in sample sizes in the CPF and CD-only groups; however, nonparametric tests were used to mitigate this imbalance. There is a potential for selection bias that is reflected in the unbalanced representation from different regions, as most patients were from Italy and Spain. In addition, most respondents (64.9%) reported having CPF, which is notably higher than the estimated cumulative incidence of CPF of 26%–28% over 20 years.^4,36^ This could potentially lead to an overestimate of the impact of CPF in CD, as the study results may not fully reflect the experiences of the CD patient population seen in general clinical practice. Furthermore, the distribution of the survey on a digital platform resulted in a selective sample of patients with internet access and technical skills; therefore, paper-based administration of the survey in nondigital contexts is needed to establish a more representative clinical sample. There is also the potential for recall bias that may have influenced some responses owing to the retrospective nature of the survey. Bias may also have been introduced due to a lack of information or understanding of treatment options for CD and CPF, or due to differences in the standard of care across the included countries. Lastly, as the current study suggests that employment status may mediate the impact of CPF on subjective well-being, this relationship warrants further investigation.

## Conclusions

This patient-reported survey illustrates that CPF places an additional burden on patients with CD by impacting overall QoL, relationships, and social and work life. In clinical practice, a multidisciplinary approach should specifically tailor therapeutic strategies to address the additional burden experienced by patients with CPF. In the future, the current survey could be used in clinical practice as a baseline measure of CPF activity to facilitate treatment selection and to monitor the effectiveness of interventions.

## Supplementary Material

otad036_suppl_Supplementary_Material

## Data Availability

The dataset will be made available to researchers who provide a methodologically sound proposal. The data will be provided after its de-identification, in compliance with applicable privacy laws, data protection, and requirements for consent and anonymization.
