# Supplementary material for: The Impact of Crohn’s Perianal Fistula on Quality of Life: Results of an International Patient Survey
Source: Crohns Colitis 360. 2023 Jul 25;5(3):otad036. doi: 10.1093/crocol/otad036 (PMC10390083; doi:10.1093/crocol/otad036)
Supplement: otad036_suppl_Supplementary_Material [file otad036_suppl_supplementary_material.docx]

**SUPPLEMENTARY INFORMATION**

**Supplementary Table S1.** Demography, disease characteristics, and diagnosis and treatment patterns of CPF stratified by gender.

|  | Female  (*n*=352) | Male  (*n*=180) |
| --- | --- | --- |
| **Country or residence**, *n* (%) |  |  |
| Italy | 85 (24.1) | 48 (26.7) |
| Spain | 85 (24.1) | 37 (20.6) |
| Portugal | 37 (10.5) | 18 (10.0) |
| Greece | 30 (8.5) | 22 (12.2) |
| Poland | 27 (7.7) | 13 (7.2) |
| Slovenia | 23 (6.5) | 7 (3.9) |
| France | 12 (3.4) | 5 (2.8) |
| Romania | 10 (2.8) | 6 (3.3) |
| Belgium | 7 (2.0) | 1 (0.6) |
| Austria | 7 (2.0) | 2 (1.1) |
| Other countries | 29 (8.2) | 21 (11.7) |
| **Age**, median (IQR) | 39.5 (32.0, 48.0) | 40.0 (32.0, 50.0) |
| **Current CD situation**, *n* (%) |  |  |
| Not active | 198 (56.3) | 109 (60.6) |
| Active | 146 (41.5) | 71 (39.4) |
| Don’t know | 8 (2.3) | 0 (0.0) |
| **CD duration**, years, *n* (%) |  |  |
| <1 | 22 (6.3) | 9 (5.0) |
| 1–5 | 76 (21.6) | 38 (21.1) |
| 6–10 | 56 (15.9) | 32 (17.8) |
| 11–15 | 48 (13.6) | 29 (16.1) |
| >15 | 150 (42.6) | 72 (40.0) |
| **Diagnosis by specialty**, *n* (%) |  |  |
| Gastroenterologist | 263 (74.7) | 148 (82.2) |
| Family doctor | 6 (1.7) | 7 (3.9) |
| Surgeon | 49 (13.9) | 19 (10.6) |
| Other physicians | 34 (9.7) | 6 (3.3) |

| **Current treatment specialty**, *n* (%) |  |  |
| --- | --- | --- |

| Family doctor | 48 (13.6) | 14 (7.8) |
| --- | --- | --- |
| Gastroenterologist | 308 (87.5) | 174 (96.7) |
| Surgeon | 132 (37.5) | 59 (32.8) |
| Other physicians | 43 (12.2) | 13 (7.2) |
| **Level of importance doctor places on treatment**, *n* (%) |  |  |
| Very high importance | 144 (40.9) | 76 (42.2) |
| Quite high importance | 112 (31.8) | 56 (31.1) |
| Some importance | 67 (19.0) | 29 (16.1) |
| Limited importance | 20 (5.7) | 13 (7.2) |
| No importance | 8 (2.3) | 3 (1.7) |
| Don’t know | 1 (0.3) | 3 (1.7) |
| **How well-informed are you about CD? ^a^** Median (IQR) | 8.00 (7.00, 9.00) | 8.00 (7.00, 9.00) |

Abbreviations: CD, Crohn’s disease; CPF, Crohn’s perianal fistula; IQR, interquartile range.

Note: All the variables include self-reported information.
Other countries include Colombia, the Dominican Republic, Greenland, Guadeloupe, Israel, Mexico, and the United States of America.
**^a^** Based on a Likert scale of 1 to 10; 1 indicating “not informed at all” and 10, “extremely well-informed.”

**Supplementary Table S2.** Current CPF symptoms stratified by gender.

| Symptoms, *n* (%) | Female (*n*=352) | Male  (*n*=180) |
| --- | --- | --- |
| **Stomach cramps** | 128 (36.4) | 52 (28.9) |
| **Diarrhea** | 137 (38.9) | 62 (34.4) |
| **Rectal bleeding** | 61 (17.3) | 28 (15.6) |
| **Loss of appetite** | 50 (14.2) | 21 (11.7) |
| **Weight loss** | 58 (16.5) | 33 (18.3) |
| **Fever** | 30 (8.5) | 16 (8.9) |
| **Joint pain** | 195 (55.4) | 72 (40.0) |
| **Fatigue** | 242 (68.8) | 90 (50.0) |
| **Pain in the anal area** | 163 (46.3) | 73 (40.6) |
| **Perianal area leakage** | 111 (31.5) | 60 (33.3) |
| **None of the above** | 36 (10.2) | 31 (17.2) |
| **Frequency of visit to physician** |  |  |
| Weekly | 24 (6.8) | 7 (3.9) |
| Monthly | 59 (16.8) | 23 (12.8) |
| Every 2–3 months | 66 (18.8) | 35 (19.4) |
| Every 4–12 months | 84 (23.9) | 51 (28.3) |
| Every 1–2 years | 53 (15.1) | 26 (14.4) |
| Every 3–4 years | 27 (7.7) | 17 (9.4) |
| Every 5 or more years | 39 (11.1) | 21 (11.7) |

Abbreviations: CPF, Crohn’s perianal fistula.

**Supplementary Table S3.** Overall impact of CPF stratified by gender.

| Survey outcome, median (IQR) | N | Female  (*n*=352) | Male  (*n*=180) |
| --- | --- | --- | --- |
| **Subjective well-being** | 532 | 7.00 (5.00, 9.00) | 7.00 (4.00, 8.00) |
| **Relationships with family/friends** | 532 | 6.00 (4.00, 8.00) | 5.00 (2.75, 7.00) |
| **Relationship with partner** | 421 ^a^ | 6.00 (3.00, 8.00) | 4.00 (2.00, 6.00) |
| **Social life** | 532 | 6.00 (4.00, 8.00) | 5.00 (2.00, 7.25) |
| **Work life** | 359 ^b^ | 7.00 (4.00, 8.00) | 5.00 (3.00, 8.00) |

Abbreviations: CPF, Crohn’s perianal fistula; IQR, interquartile range.

^a^ *n*=111 respondents reported not being in a relationship with a partner.
^b^ *n*=173 respondents were not employed.
Based on a Likert scale of 1 to 10; 1 indicating “no impact at all” and 10, “very big impact.”
N indicates patients with CPF only.

**Supplementary Figure S1**Survey structure

**
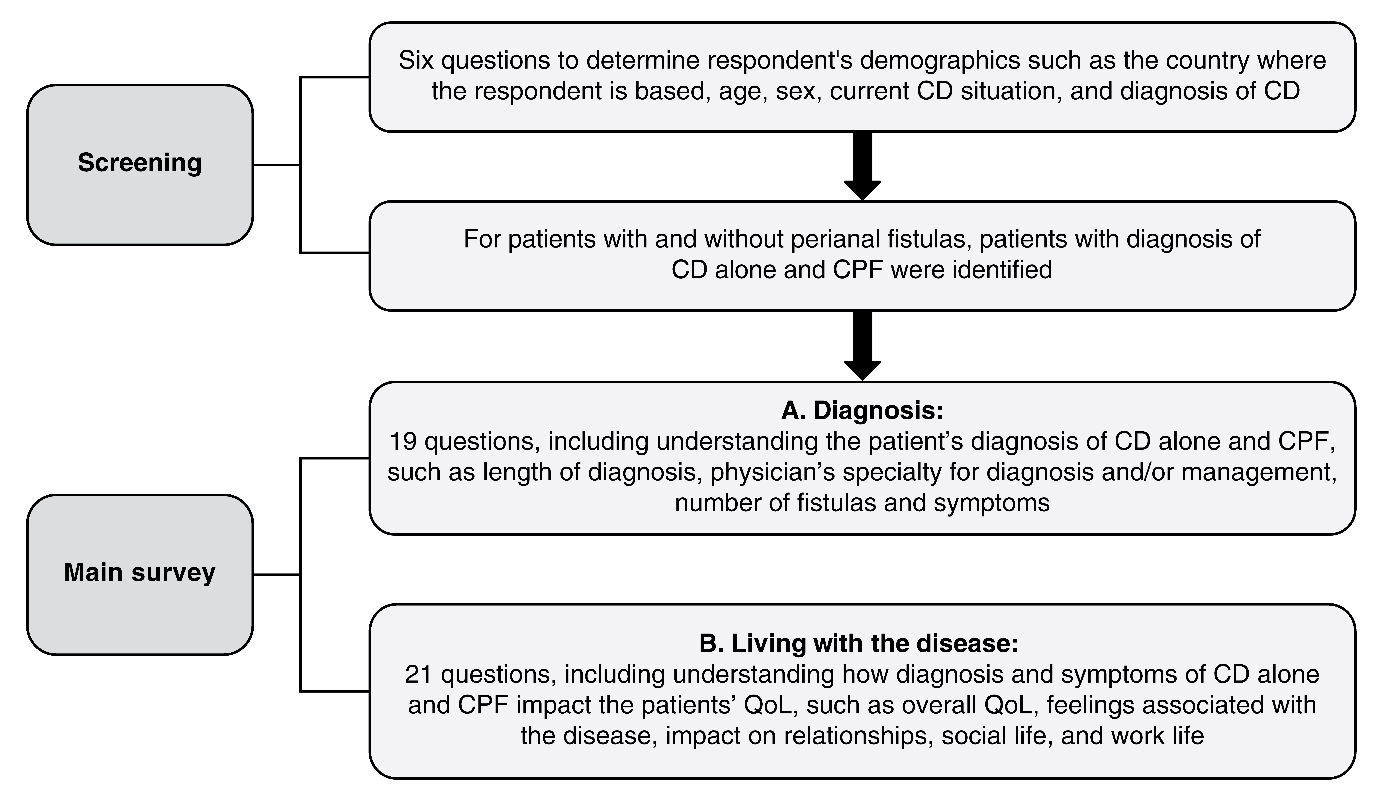
**

Abbreviations: CD, Crohn’s disease; CPF, Crohn’s perianal fistula; QoL, quality of life.

**Supplementary Figure S2** Impact of Crohn’s perianal fistula stratified by gender. (A) Subjective well-being. (B) Relationship with friends and family. (C) Relationship with partner (number of respondents for relationship with partner is based on presence of partner and presence of fistula; men, n=146 and women, n=275). (D) Social life. (E) Work life.
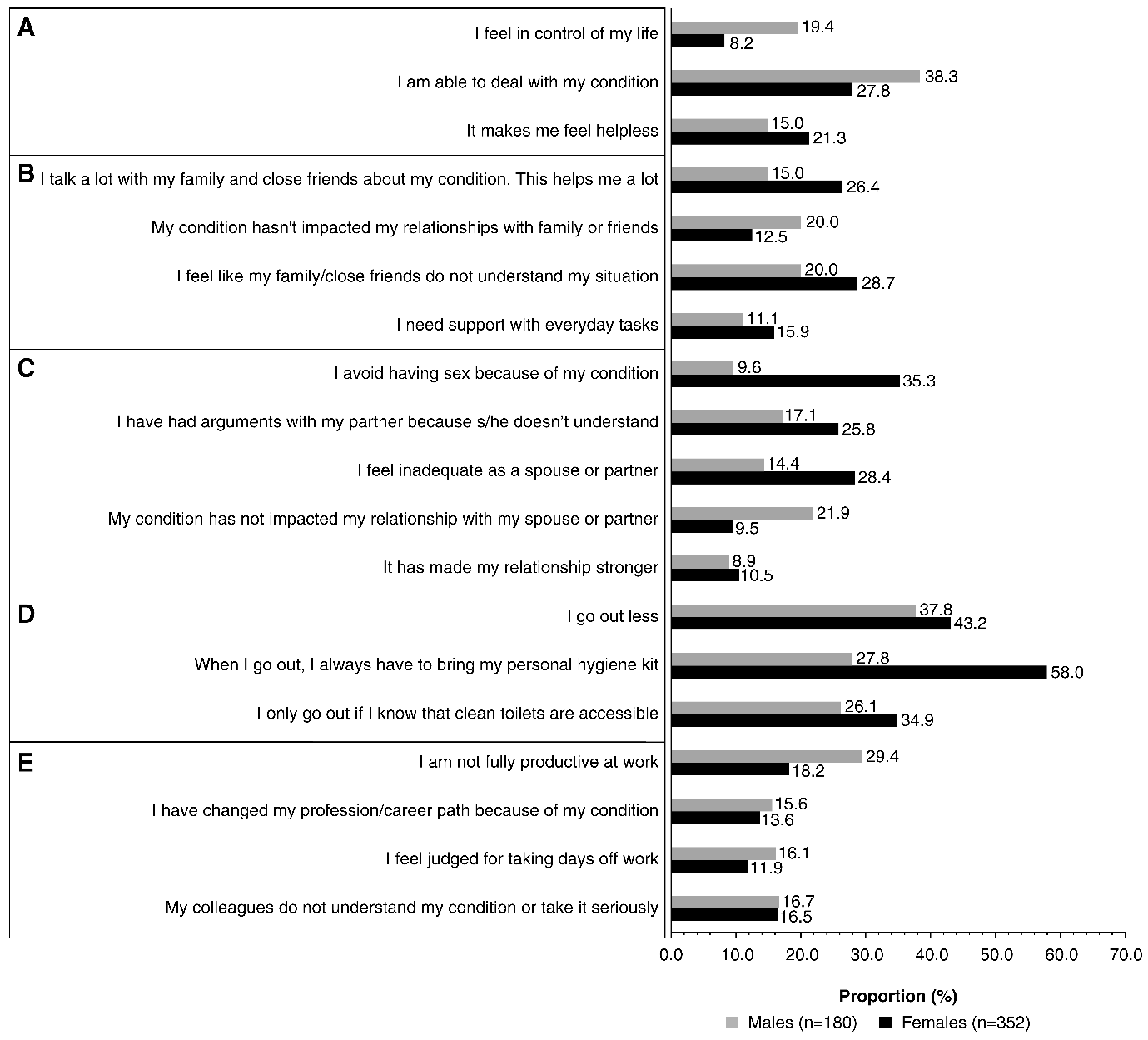


Abbreviations: CPF, Crohn’s perianal fistula.

**Supplementary Data 1**

**International patient survey**

**Questionnaire to understand the quality of life of patients diagnosed with Crohn’s disease only or Crohn’s perianal fistula**

PART General

**Question 1**

Country where the respondent is based:

| A1 | Afghanistan | A62 | Equatorial Guinea | A123 | Madagascar | A184 | | Samoa |
| --- | --- | --- | --- | --- | --- | --- | --- | --- |
| A2 | Albania | A63 | Eritrea | A124 | Malawi | A185 | | San Marino |
| A3 | Algeria | A64 | Estonia | A125 | Malaysia | A186 | | Sao Tome and Principe |
| A4 | American Samoa | A65 | Ethiopia | A126 | Maldives | A187 | | Saudi Arabia |
| A5 | Andorra | A66 | Falkland Islands | A127 | Mali | A188 | | Senegal |
| A6 | Angola | A67 | Faroe Islands | A128 | Malta | A189 | | Serbia |
| A7 | Anguilla | A68 | Fiji | A129 | Marshall Islands | A190 | | Seychelles |
| A8 | Antigua and Barbuda | A69 | Finland | A130 | Martinique | A191 | | Sierra Leone |
| A9 | Argentina | A70 | France | A131 | Mauritania | A192 | | Singapore |
| A10 | Armenia | A71 | French Polynesia | A132 | Mauritius | A193 | | Slovakia |
| A11 | Aruba | A72 | Gabon | A133 | Mayotte | A194 | | Slovenia |
| A12 | Australia | A73 | The Gambia | A134 | Mexico | A195 | | Solomon Islands |
| A13 | Austria | A74 | Georgia | A135 | Micronesia | A196 | | Somalia |
| A14 | Azerbaijan | A75 | Germany | A136 | Moldova | A197 | | Somaliland |
| A15 | The Bahamas | A76 | Ghana | A137 | Monaco | A198 | | South Africa |
| A16 | Bahrain | A77 | Gibraltar | A138 | Mongolia | A199 | | South Ossetia |
| A17 | Bangladesh | A78 | Greece | A139 | Montenegro | A200 | | Spain |
| A18 | Barbados | A79 | Greenland | A140 | Montserrat | A201 | | Sri Lanka |
| A19 | Belarus | A80 | Grenada | A141 | Morocco | A202 | | Sudan |
| A20 | Belgium | A81 | Guadeloupe | A142 | Mozambique | A203 | | Suriname |
| A21 | Belize | A82 | Guam | A143 | Myanmar | A204 | | Svalbard |
| A22 | Benin | A83 | Guatemala | A144 | Nagorno - Karabakh | A205 | | Swaziland |
| A23 | Bermuda | A84 | Guernsey | A145 | Namibia | A206 | | Sweden |
| A24 | Bhutan | A85 | Guinea | A146 | Nauru | A207 | | Switzerland |
| A25 | Bolivia | A86 | Guinea - Bissau | A147 | Nepal | A208 | | Syria |
| A26 | Bosnia and Herzegovina | A87 | Guyana | A148 | Netherlands | A209 | | Taiwan |
| A27 | Botswana | A88 | Haiti | A149 | Netherlands Antilles | A210 | | Tajikistan |
| A28 | Brazil | A89 | Honduras | A150 | New Caledonia | A211 | | Tanzania |
| A29 | Brunei | A90 | Hong Kong | A151 | New Zealand | A212 | | Thailand |
| A30 | Bulgaria | A91 | Hungary | A152 | Nicaragua | A213 | | Timor-Leste |
| A31 | Burkina Faso | A92 | Iceland | A153 | Niger | A214 | | Togo |
| A32 | Burundi | A93 | India | A154 | Nigeria | A215 | | Tokelau |
| A33 | Cambodia | A94 | Indonesia | A155 | Niue | A216 | | Tonga |
| A34 | Cameroon | A95 | Iran | A156 | Norfolk Island | A217 | | Transnistria Pridnestrovie |
| A35 | Canada | A96 | Iraq | A157 | Turkish Republic of Northern Cyprus | A218 | | Trinidad and Tobago |
| A36 | Cape Verde | A97 | Ireland | A158 | Northern Mariana | A219 | | Tristan da Cunha |
| A37 | Cayman Islands | A98 | Israel | A159 | Norway | A220 | | Tunisia |
| A38 | Central African Republic | A99 | Italy | A160 | Oman | A221 | | Turkey |
| A39 | Chad | A100 | Jamaica | A161 | Pakistan | A222 | | Turkmenistan |
| A40 | Chile | A101 | Japan | A162 | Palau | A223 | | Turks and Caicos Islands |
| A41 | People’s Republic of China | A102 | Jersey | A163 | Palestine | A224 | | Tuvalu |
| A42 | Republic of China | A103 | Jordan | A164 | Panama | A225 | | Uganda |
| A43 | Christmas Island | A104 | Kazakhstan | A165 | Papua New Guinea | A226 | | Ukraine |
| A44 | Cocos (Keeling) Islands | A105 | Kenya | A166 | Paraguay | A227 | | United Arab Emirates |
| A45 | Colombia | A106 | Kiribati | A167 | Peru | A228 | | United Kingdom |
| A46 | Comoros | A107 | North Korea | A168 | Philippines | A229 | | United States |
| A47 | Congo | A108 | South Korea | A169 | Pitcairn Islands | A230 | | Uruguay |
| A48 | Cook Islands | A109 | Kosovo | A170 | Poland | A231 | | Uzbekistan |
| A49 | Costa Rica | A110 | Kuwait | A171 | Portugal | A232 | | Vanuatu |
| A50 | Cote d’Ivoire | A111 | Kyrgyzstan | A172 | Puerto Rico | A233 | | Vatican City |
| A51 | Croatia | A112 | Laos | A173 | Qatar | A234 | | Venezuela |
| A52 | Cuba | A113 | Latvia | A174 | Romania | A235 | | Vietnam |
| A53 | Cyprus | A114 | Lebanon | A175 | Russia | A236 | | British Virgin Islands |
| A54 | Czech Republic | A115 | Lesotho | A176 | Rwanda | A237 | | Isle of Man |
| A55 | Denmark | A116 | Liberia | A177 | Saint Barthelemy | A238 | | US Virgin Islands |
| A56 | Djibouti | A117 | Libya | A178 | Saint Helena | A239 | | Wallis and Futuna |
| A57 | Dominica | A118 | Liechtenstein | A179 | Saint Kitts and Nevis | A240 | | Western Sahara |
| A58 | Dominican Republic | A119 | Lithuania | A180 | Saint Lucia | A241 | | Yemen |
| A59 | Ecuador | A120 | Luxembourg | A181 | Saint Martin | A242 | | Zambia |
| A60 | Egypt | A121 | Macau | A182 | Saint Pierre and Miquelon | A243 | | Zimbabwe |
| A61 | El Salvador | A122 | Macedonia | A183 | Saint Vincent and the Grenadines | |  |  |

**Question 2**

Please enter your age.

(write a number between 10 and 100)

**Question 3**

Please indicate your gender.

(MALE or FEMALE)

**Question 4**

Have you been diagnosed with Crohn’s disease?

A1 Yes

A2 No

A3 Don’t know

**Question 5**

Please read through the following statements and tick the one that fits your situation best.

A1 My Crohn’s disease is currently not active - I am experiencing no / mild symptoms

A2 My Crohn’s disease is currently active - I am experiencing moderate to severe symptoms

A3 Don’t know

**Question 6**

Have you been diagnosed with Crohn’s disease AND perianal fistula (fistula in the anal area)?

A1 Yes

A2 No

A3 Don’t know

PART Diagnosis

**Question 1**

How long has it been since your *Crohn’s disease* diagnosis?

A1 Less than 1 year

A2 1–5 years

A3 6–10 years

A4 11–15 years

A5 More than 15 years

**Question 2**

What type of physician diagnosed you with *Crohn’s disease*?

A1 General Practitioner / Family Doctor

A2 Gastroenterologist

A3 Surgeon

A4 Other type of physician, please specify:

**Question 3**

What type of physicians are responsible for currently managing your *Crohn’s disease* or *Crohn’s disease–related perianal fistula*? Please tick all that apply.

A1 General Practitioner / Family Doctor

A2 Gastroenterologist

A3 Surgeon

A4 Other type of physician, please specify:

**Question 4**

Thinking of the physician who is currently mainly responsible for managing your *Crohn’s disease*, how much importance do you feel the doctor places on your condition?

A1 I feel that the doctor **places a very high level of importance** on my Crohn’s disease

A2 I feel that the doctor **places a quite high level of importance** on my Crohn’s disease

A3 I feel that the doctor **places some level of importance** on my Crohn’s disease

A4 I feel that the doctor **places a limited level of importance** on my Crohn’s disease

A5 I feel that the doctor **places no importance** on my Crohn’s disease

A6 Don’t know

**Question 5**

On a scale from 1 to 10, where 1=not informed at all, and 10=extremely well-informed, how well-informed do you think you are about your *Crohn’s disease?* Please tick.

| A1 | 1 | A6 | 6 |
| --- | --- | --- | --- |
| A2 | 2 | A7 | 7 |
| A3 | 3 | A8 | 8 |
| A4 | 4 | A9 | 9 |
| A5 | 5 | A10 | 10 |

**Question 6**

How long has it been since your *perianal fistula* has been diagnosed?

A1 Less than 1 year

A2 1–5 years

A3 6–10 years

A4 11–15 years

A5 More than 15 years

**Question 7**

What type of physician diagnosed your *perianal fistula*?

A1 General Practitioner / Family Doctor

A2 Gastroenterologist

A3 Specialized surgeon

A4 Other type of physician, please specify:

**Question 8**

What type of physicians are responsible for currently managing your *perianal fistula*? Please tick all that apply.

A1 General Practitioner / Family Doctor

A2 Gastroenterologist

A3 Surgeon

A4 Other type of physician, please specify:

**Question 9**

Thinking of the physician who is currently mainly responsible for managing your perianal fistulas, how much importance do you feel the doctor places on your condition?

A1 I feel that the doctor **places a very high level of importance** on my perianal fistulas

A2 I feel that the doctor **places a quite high level of importance** on my perianal fistulas

A3 I feel that the doctor **places some level of importance** on my perianal fistulas

A4 I feel that the doctor **places a limited level of importance** on my perianal fistulas

A5 I feel that the doctor **places no importance** on my perianal fistulas

A6 Don’t know

**Question 10**

On a scale from 1 to 10, where 1=not informed at all, and 10=extremely well-informed, how well-informed do you think you are about your *perianal fistula*? Please tick.

| A1 | 1 | A6 | 6 |
| --- | --- | --- | --- |
| A2 | 2 | A7 | 7 |
| A3 | 3 | A8 | 8 |
| A4 | 4 | A9 | 9 |
| A5 | 5 | A10 | 10 |

**Question 11**

Have you ever been told that your perianal fistula is COMPLEX?

A1 Yes

A2 No

A3 Can’t remember

**Question 12**

How many perianal fistulas are you currently suffering from?

(write a number between 1 and 100)

**Question 13**

Do you have rectovaginal fistula?

(YES or NO)

**Question 14**

Which of the following symptoms of *Crohn’s disease* have you ever experienced? Please select all that apply.

| A1 | Painful stomach cramps | A7 | Joint pain |
| --- | --- | --- | --- |
| A2 | Persistent or recurrent diarrhea | A8 | Fatigue |
| A3 | Rectal bleeding | A9 | Pain in / around the anal area |
| A4 | Loss of appetite | A10 | Leaking / drainage in the perianal area |
| A5 | Weight loss | A11 | None of the above |
| A6 | Fever |  |  |

**Question 15**

Which of the following symptoms of *Crohn’s disease are you currently* experiencing? Please select all that apply.

| A1 | Painful stomach cramps | A7 | Joint pain |
| --- | --- | --- | --- |
| A2 | Persistent or recurrent diarrhea | A8 | Fatigue |
| A3 | Rectal bleeding | A9 | Pain in / around the anal area |
| A4 | Loss of appetite | A10 | Leaking / drainage in perianal area |
| A5 | Weight loss | A11 | None of the above |
| A6 | Fever |  |  |

**Question 16**

How often do you experience symptom flare-ups of your *Crohn’s disease* that lead to a physician visit?

| A1 | Weekly | A5 | Every 1–2 years |
| --- | --- | --- | --- |
| A2 | Monthly | A6 | Every 3–4 years |
| A3 | Every 2–3 months | A7 | Every 5 or more years |
| A4 | Every 4–12 months |  | |

**Question 17**

Which of the following symptoms have you ever experienced due to your *perianal fistulas*? Please select all that apply.

| A1 | Pain | A6 | Foul-smelling liquid oozing |
| --- | --- | --- | --- |
| A2 | Redness in the anal area | A7 | Purulent oozing |
| A3 | Swelling of the anal area | A8 | Fever |
| A4 | Bleeding | A9 | Fatigue |
| A5 | Itching | A10 | None of the above |

**Question 18**

Which of the following symptoms are you currently experiencing due to your *perianal fistulas*? Please select all that apply.

| A1 | Pain | A6 | Foul-smelling liquid oozing |
| --- | --- | --- | --- |
| A2 | Redness in the anal area | A7 | Purulent oozing |
| A3 | Swelling of the anal area | A8 | Fever |
| A4 | Bleeding | A9 | Fatigue |
| A5 | Itching | A10 | None of the above |

**Question 19**

How often do you experience symptom flare-ups of your *perianal fistula* that lead to a physician visit?

| A1 | Weekly | A5 | Every 1–2 years |
| --- | --- | --- | --- |
| A2 | Monthly | A6 | Every 3–4 years |
| A3 | Every 2–3 months | A7 | Every 5 or more years |
| A4 | Every 4–12 months |  | |

PART Living with the disease

**Question 1**

On a scale from 1 to 10, where 1=No impact at all, and 10=Very big impact, what is currently the **impact of your condition on your overall quality of life**. (eg, overall well-being, your ability to perform daily life activities, and the extent to which your life is satisfying in terms of health and happiness). Please tick.

| A1 | 1 | A6 | 6 |
| --- | --- | --- | --- |
| A2 | 2 | A7 | 7 |
| A3 | 3 | A8 | 8 |
| A4 | 4 | A9 | 9 |
| A5 | 5 | A10 | 10 |

**Question 2**

How does living with your condition make you feel? Please select all that apply.

| A1 | It makes me feel depressed | A11 | I feel ashamed of my body |
| --- | --- | --- | --- |
| A2 | It makes me feel unattractive | A12 | I feel that my condition has taken over my life |
| A3 | It makes me feel helpless | A13 | Some days my condition is so bad, I hate my life |
| A4 | It makes me feel anxious | A14 | It has no impact on how I feel about myself |
| A5 | It makes me feel like I am not normal or different | A15 | I feel self-conscious of my body |
| A6 | It makes me feel dirty | A16 | It makes me feel empowered |
| A7 | It makes me feel uncomfortable | A17 | I am able to deal with my condition |
| A8 | It makes me feel less confident about myself | A18 | I feel in control of my life |
| A9 | I feel other people are judging me | A19 | None of the above |
| A10 | I feel other people do not understand my condition and the implications |  | |

**Question 3**

On a scale from 1 to 10, where 1=No impact at all, and 10=Very big impact, what is currently the **impact of your condition on your relationships with family or close friends?** Please tick.

| A1 | 1 | A6 | 6 |
| --- | --- | --- | --- |
| A2 | 2 | A7 | 7 |
| A3 | 3 | A8 | 8 |
| A4 | 4 | A9 | 9 |
| A5 | 5 | A10 | 10 |

**Question 4**

In what ways does your condition impact your **relationships?** Please select all that apply.

| A1 | It made me closer to my family / friends | A9 | I feel like my family / close friends are very understanding |
| --- | --- | --- | --- |
| A2 | It made me more distant to my family / friends | A10 | My condition hasn’t impacted my relationships with family or friends |
| A3 | I avoid making commitments | A11 | Because of my condition, I do not have a close circle of family/ friends |
| A4 | I need support with everyday tasks | A12 | It has made my relationship to family or friends stronger |
| A5 | I have missed out on family events or gatherings, such as birthdays or weddings | A13 | I talk a lot with my family and close friends about my condition. This helps me a lot |
| A6 | I feel guilty about the impact of my condition on my family / friends | A14 | It is difficult to talk with my family and close friends about my condition |
| A7 | I feel like my family / close friends do not understand my situation | A15 | I avoid talking with my family and friends about my condition |
| A8 | I feel judged | A16 | None of the above |

**Question 5**

On a scale from 1 to 10, where 1=No impact at all, and 10=Very big impact, what is currently the **impact of your condition on your relationships with a spouse or partner?** Please tick.

| A1 | 1 | A7 | 7 |
| --- | --- | --- | --- |
| A2 | 2 | A8 | 8 |
| A3 | 3 | A9 | 9 |
| A4 | 4 | A10 | 10 |
| A5 | 5 | A11 | I am currently not in a relationship with a partner or spouse |
| A6 | 6 |  | |

**Question 6**

In what ways does your condition impact **your past or current relationships with a spouse or partner?** Please select all that apply.

| A1 | I avoid having sex because of my condition | A10 | It has made my relationship stronger |
| --- | --- | --- | --- |
| A2 | A partner has ended a relationship with me | A11 | My partner has refused to have sex with me |
| A3 | I avoid having intimate relationships with people | A12 | My partner loves me just the way I am |
| A4 | I have had arguments with my partner because s/he doesn’t understand | A13 | My partner is very understanding |
| A5 | I feel inadequate as a spouse or partner | A14 | My partner and I talk a lot about my condition. This helps me a lot |
| A6 | I can’t stand the thought of someone touching me | A15 | It is difficult to talk with my partner about my condition |
| A7 | Partners have been disgusted or repulsed by my condition | A16 | My partner and I avoid talking about my condition |
| A8 | I avoid dating because I am ashamed of my condition | A17 | None of the above |
| A9 | My condition has not impacted my relationship with my spouse or partner |  | |

**Question 7**

Please select the level of restriction of sexual activity that you are experiencing due to your condition.

A1 No restriction in sexual activity

A2 Slight restriction in sexual activity

A3 Moderate restriction in sexual activity

A4 Marked limitation in sexual activity

A5 Unable to engage in sexual activity

**Question 8**

On a scale from 1 to 10, where 1=No impact at all, and 10=Very big impact, what is currently the **impact of your condition on your social life?** By this we mean activities with your wider network such as going out with friends. Please tick.

| A1 | 1 | A6 | 6 |
| --- | --- | --- | --- |
| A2 | 2 | A7 | 7 |
| A3 | 3 | A8 | 8 |
| A4 | 4 | A9 | 9 |
| A5 | 5 | A10 | 10 |

**Question 9**

In what ways does your condition impact **your social life?** Please select all that apply.

| A1 | I cannot take part in all activities / hobbies that I used to | A8 | When I go out, I do not stay out for a very long time |
| --- | --- | --- | --- |
| A2 | I cannot take part in sports / exercise | A9 | When I go out, I always have to bring my personal hygiene kit |
| A3 | It has stopped me from going on holidays / vacation | A10 | I only go out if I know that clean toilets are accessible |
| A4 | It has stopped me from going to social events | A11 | I go out less |
| A5 | It has stopped me from going to restaurants | A12 | My condition does not hold me back |
| A6 | It has stopped me from going to the cinema | A13 | Because of my condition, I am socially isolated |
| A7 | I do not invite friends to my house | A14 | None of the above |

**Question 10**

Please specify your main occupation status.

| A1 | Full-time employed | A5 | Retired |
| --- | --- | --- | --- |
| A2 | Part-time employed | A6 | Student |
| A3 | Unemployed | A7 | Home maker / housewife |
| A4 | Self-employed | A8 | Other |

**Question 11**

Please describe your work experiences in the past month. These experiences may be affected by many environmental as well as personal factors and may change from time to time. For each of the following statements, please check one of the following responses to show your agreement or disagreement with this statement in describing your work experiences in the past month. “Because of my condition, the stresses of my job were much harder to handle."”

A1 1 Strongly disagree

A2 2 Somewhat disagree

A3 3 Uncertain

A4 4 Somewhat agree

A5 5 Strongly agree

**Question 12**

Please describe your work experiences in the past month. These experiences may be affected by many environmental as well as personal factors and may change from time to time. For each of the following statements, please check one of the following responses to show your agreement or disagreement with this statement in describing your work experiences in the past month. “Despite having my condition, I was able to finish hard tasks in my work."”

A1 1 Strongly disagree

A2 2 Somewhat disagree

A3 3 Uncertain

A4 4 Somewhat agree

A5 5 Strongly agree

**Question 13**

Please describe your work experiences in the past month. These experiences may be affected by many environmental as well as personal factors and may change from time to time. For each of the following statements, please check one of the following responses to show your agreement or disagreement with this statement in describing your work experiences in the past month. “My condition distracted me from taking pleasure in my work.”

A1 1 Strongly disagree

A2 2 Somewhat disagree

A3 3 Uncertain

A4 4 Somewhat agree

A5 5 Strongly agree

**Question 14**

Please describe your work experiences in the past month. These experiences may be affected by many environmental as well as personal factors and may change from time to time. For each of the following statements, please check one of the following responses to show your agreement or disagreement with this statement in describing your work experiences in the past month. “I felt hopeless about finishing certain work tasks, due to my condition.”

A1 1 Strongly disagree

A2 2 Somewhat disagree

A3 3 Uncertain

A4 4 Somewhat agree

A5 5 Strongly agree

**Question 15**

Please describe your work experiences in the past month. These experiences may be affected by many environmental as well as personal factors and may change from time to time. For each of the following statements, please check one of the following responses to show your agreement or disagreement with this statement in describing your work experiences in the past month. “At work, I was able to focus on achieving my goals despite my condition.”

A1 1 Strongly disagree

A2 2 Somewhat disagree

A3 3 Uncertain

A4 4 Somewhat agree

A5 5 Strongly agree

**Question 16**

Please describe your work experiences in the past month. These experiences may be affected by many environmental as well as personal factors and may change from time to time. For each of the following statements, please check one of the following responses to show your agreement or disagreement with this statement in describing your work experiences in the past month. “Despite having my condition, I felt energetic enough to complete all my work.”

A1 1 Strongly disagree

A2 2 Somewhat disagree

A3 3 Uncertain

A4 4 Somewhat agree

A5 5 Strongly agree

**Question 17**

How many days, if any, in the last six months have you had off work because of your *perianal fistulas*?

| A1 | 0 | A52 | 51 | A103 | 102 | A154 | 153 |
| --- | --- | --- | --- | --- | --- | --- | --- |
| A2 | 1 | A53 | 52 | A104 | 103 | A155 | 154 |
| A3 | 2 | A54 | 53 | A105 | 104 | A156 | 155 |
| A4 | 3 | A55 | 54 | A106 | 105 | A157 | 156 |
| A5 | 4 | A56 | 55 | A107 | 106 | A158 | 157 |
| A6 | 5 | A57 | 56 | A108 | 107 | A159 | 158 |
| A7 | 6 | A58 | 57 | A109 | 108 | A160 | 159 |
| A8 | 7 | A59 | 58 | A110 | 109 | A161 | 160 |
| A9 | 8 | A60 | 59 | A111 | 110 | A162 | 161 |
| A10 | 9 | A61 | 60 | A112 | 111 | A163 | 162 |
| A11 | 10 | A62 | 61 | A113 | 112 | A164 | 163 |
| A12 | 11 | A63 | 62 | A114 | 113 | A165 | 164 |
| A13 | 12 | A64 | 63 | A115 | 114 | A166 | 165 |
| A14 | 13 | A65 | 64 | A116 | 115 | A167 | 166 |
| A15 | 14 | A66 | 65 | A117 | 116 | A168 | 167 |
| A16 | 15 | A67 | 66 | A118 | 117 | A169 | 168 |
| A17 | 16 | A68 | 67 | A119 | 118 | A170 | 169 |
| A18 | 17 | A69 | 68 | A120 | 119 | A171 | 170 |
| A19 | 18 | A70 | 69 | A121 | 120 | A172 | 171 |
| A20 | 19 | A71 | 70 | A122 | 121 | A173 | 172 |
| A21 | 20 | A72 | 71 | A123 | 122 | A174 | 173 |
| A22 | 21 | A73 | 72 | A124 | 123 | A175 | 174 |
| A23 | 22 | A74 | 73 | A125 | 124 | A176 | 175 |
| A24 | 23 | A75 | 74 | A126 | 125 | A177 | 176 |
| A25 | 24 | A76 | 75 | A127 | 126 | A178 | 177 |
| A26 | 25 | A77 | 76 | A128 | 127 | A179 | 178 |
| A27 | 26 | A78 | 77 | A129 | 128 | A180 | 179 |
| A28 | 27 | A79 | 78 | A130 | 129 | A181 | 180 |
| A29 | 28 | A80 | 79 | A131 | 130 | A182 | 181 |
| A30 | 29 | A81 | 80 | A132 | 131 | A183 | 182 |
| A31 | 30 | A82 | 81 | A133 | 132 | A184 | 183 |
| A32 | 31 | A83 | 82 | A134 | 133 | A185 | 184 |
| A33 | 32 | A84 | 83 | A135 | 134 | A186 | 185 |
| A34 | 33 | A85 | 84 | A136 | 135 | A187 | 186 |
| A35 | 34 | A86 | 85 | A137 | 136 | A188 | 187 |
| A36 | 35 | A87 | 86 | A138 | 137 | A189 | 188 |
| A37 | 36 | A88 | 87 | A139 | 138 | A190 | 189 |
| A38 | 37 | A89 | 88 | A140 | 139 | A191 | 190 |
| A39 | 38 | A90 | 89 | A141 | 140 | A192 | 191 |
| A40 | 39 | A91 | 90 | A142 | 141 | A193 | 192 |
| A41 | 40 | A92 | 91 | A143 | 142 | A194 | 193 |
| A42 | 41 | A93 | 92 | A144 | 143 | A195 | 194 |
| A43 | 42 | A94 | 93 | A145 | 144 | A196 | 195 |
| A44 | 43 | A95 | 94 | A146 | 145 | A197 | 196 |
| A45 | 44 | A96 | 95 | A147 | 146 | A198 | 197 |
| A46 | 45 | A97 | 96 | A148 | 147 | A199 | 198 |
| A47 | 46 | A98 | 97 | A149 | 148 | A200 | 199 |
| A48 | 47 | A99 | 98 | A150 | 149 | A201 | 200 |
| A49 | 48 | A100 | 99 | A151 | 150 |  |  |
| A50 | 49 | A101 | 100 | A152 | 151 |  |  |
| A51 | 50 | A102 | 101 | A153 | 152 |  |  |

**Question 18**

How many days, if any, in the last six months have you had off work because of your *Crohn’s disease*?

| A1 | 0 | A52 | 51 | A103 | 102 | A154 | 153 |
| --- | --- | --- | --- | --- | --- | --- | --- |
| A2 | 1 | A53 | 52 | A104 | 103 | A155 | 154 |
| A3 | 2 | A54 | 53 | A105 | 104 | A156 | 155 |
| A4 | 3 | A55 | 54 | A106 | 105 | A157 | 156 |
| A5 | 4 | A56 | 55 | A107 | 106 | A158 | 157 |
| A6 | 5 | A57 | 56 | A108 | 107 | A159 | 158 |
| A7 | 6 | A58 | 57 | A109 | 108 | A160 | 159 |
| A8 | 7 | A59 | 58 | A110 | 109 | A161 | 160 |
| A9 | 8 | A60 | 59 | A111 | 110 | A162 | 161 |
| A10 | 9 | A61 | 60 | A112 | 111 | A163 | 162 |
| A11 | 10 | A62 | 61 | A113 | 112 | A164 | 163 |
| A12 | 11 | A63 | 62 | A114 | 113 | A165 | 164 |
| A13 | 12 | A64 | 63 | A115 | 114 | A166 | 165 |
| A14 | 13 | A65 | 64 | A116 | 115 | A167 | 166 |
| A15 | 14 | A66 | 65 | A117 | 116 | A168 | 167 |
| A16 | 15 | A67 | 66 | A118 | 117 | A169 | 168 |
| A17 | 16 | A68 | 67 | A119 | 118 | A170 | 169 |
| A18 | 17 | A69 | 68 | A120 | 119 | A171 | 170 |
| A19 | 18 | A70 | 69 | A121 | 120 | A172 | 171 |
| A20 | 19 | A71 | 70 | A122 | 121 | A173 | 172 |
| A21 | 20 | A72 | 71 | A123 | 122 | A174 | 173 |
| A22 | 21 | A73 | 72 | A124 | 123 | A175 | 174 |
| A23 | 22 | A74 | 73 | A125 | 124 | A176 | 175 |
| A24 | 23 | A75 | 74 | A126 | 125 | A177 | 176 |
| A25 | 24 | A76 | 75 | A127 | 126 | A178 | 177 |
| A26 | 25 | A77 | 76 | A128 | 127 | A179 | 178 |
| A27 | 26 | A78 | 77 | A129 | 128 | A180 | 179 |
| A28 | 27 | A79 | 78 | A130 | 129 | A181 | 180 |
| A29 | 28 | A80 | 79 | A131 | 130 | A182 | 181 |
| A30 | 29 | A81 | 80 | A132 | 131 | A183 | 182 |
| A31 | 30 | A82 | 81 | A133 | 132 | A184 | 183 |
| A32 | 31 | A83 | 82 | A134 | 133 | A185 | 184 |
| A33 | 32 | A84 | 83 | A135 | 134 | A186 | 185 |
| A34 | 33 | A85 | 84 | A136 | 135 | A187 | 186 |
| A35 | 34 | A86 | 85 | A137 | 136 | A188 | 187 |
| A36 | 35 | A87 | 86 | A138 | 137 | A189 | 188 |
| A37 | 36 | A88 | 87 | A139 | 138 | A190 | 189 |
| A38 | 37 | A89 | 88 | A140 | 139 | A191 | 190 |
| A39 | 38 | A90 | 89 | A141 | 140 | A192 | 191 |
| A40 | 39 | A91 | 90 | A142 | 141 | A193 | 192 |
| A41 | 40 | A92 | 91 | A143 | 142 | A194 | 193 |
| A42 | 41 | A93 | 92 | A144 | 143 | A195 | 194 |
| A43 | 42 | A94 | 93 | A145 | 144 | A196 | 195 |
| A44 | 43 | A95 | 94 | A146 | 145 | A197 | 196 |
| A45 | 44 | A96 | 95 | A147 | 146 | A198 | 197 |
| A46 | 45 | A97 | 96 | A148 | 147 | A199 | 198 |
| A47 | 46 | A98 | 97 | A149 | 148 | A200 | 199 |
| A48 | 47 | A99 | 98 | A150 | 149 | A201 | 200 |
| A49 | 48 | A100 | 99 | A151 | 150 |  |  |
| A50 | 49 | A101 | 100 | A152 | 151 |  |  |
| A51 | 50 | A102 | 101 | A153 | 152 |  |  |

**Question 19**

How many days in total, if any, in the last six months have you had off work because of your *Crohn’s disease* AND your *perianal fistulas*?

| A1 | 0 | A52 | 51 | A103 | 102 | A154 | 153 |
| --- | --- | --- | --- | --- | --- | --- | --- |
| A2 | 1 | A53 | 52 | A104 | 103 | A155 | 154 |
| A3 | 2 | A54 | 53 | A105 | 104 | A156 | 155 |
| A4 | 3 | A55 | 54 | A106 | 105 | A157 | 156 |
| A5 | 4 | A56 | 55 | A107 | 106 | A158 | 157 |
| A6 | 5 | A57 | 56 | A108 | 107 | A159 | 158 |
| A7 | 6 | A58 | 57 | A109 | 108 | A160 | 159 |
| A8 | 7 | A59 | 58 | A110 | 109 | A161 | 160 |
| A9 | 8 | A60 | 59 | A111 | 110 | A162 | 161 |
| A10 | 9 | A61 | 60 | A112 | 111 | A163 | 162 |
| A11 | 10 | A62 | 61 | A113 | 112 | A164 | 163 |
| A12 | 11 | A63 | 62 | A114 | 113 | A165 | 164 |
| A13 | 12 | A64 | 63 | A115 | 114 | A166 | 165 |
| A14 | 13 | A65 | 64 | A116 | 115 | A167 | 166 |
| A15 | 14 | A66 | 65 | A117 | 116 | A168 | 167 |
| A16 | 15 | A67 | 66 | A118 | 117 | A169 | 168 |
| A17 | 16 | A68 | 67 | A119 | 118 | A170 | 169 |
| A18 | 17 | A69 | 68 | A120 | 119 | A171 | 170 |
| A19 | 18 | A70 | 69 | A121 | 120 | A172 | 171 |
| A20 | 19 | A71 | 70 | A122 | 121 | A173 | 172 |
| A21 | 20 | A72 | 71 | A123 | 122 | A174 | 173 |
| A22 | 21 | A73 | 72 | A124 | 123 | A175 | 174 |
| A23 | 22 | A74 | 73 | A125 | 124 | A176 | 175 |
| A24 | 23 | A75 | 74 | A126 | 125 | A177 | 176 |
| A25 | 24 | A76 | 75 | A127 | 126 | A178 | 177 |
| A26 | 25 | A77 | 76 | A128 | 127 | A179 | 178 |
| A27 | 26 | A78 | 77 | A129 | 128 | A180 | 179 |
| A28 | 27 | A79 | 78 | A130 | 129 | A181 | 180 |
| A29 | 28 | A80 | 79 | A131 | 130 | A182 | 181 |
| A30 | 29 | A81 | 80 | A132 | 131 | A183 | 182 |
| A31 | 30 | A82 | 81 | A133 | 132 | A184 | 183 |
| A32 | 31 | A83 | 82 | A134 | 133 | A185 | 184 |
| A33 | 32 | A84 | 83 | A135 | 134 | A186 | 185 |
| A34 | 33 | A85 | 84 | A136 | 135 | A187 | 186 |
| A35 | 34 | A86 | 85 | A137 | 136 | A188 | 187 |
| A36 | 35 | A87 | 86 | A138 | 137 | A189 | 188 |
| A37 | 36 | A88 | 87 | A139 | 138 | A190 | 189 |
| A38 | 37 | A89 | 88 | A140 | 139 | A191 | 190 |
| A39 | 38 | A90 | 89 | A141 | 140 | A192 | 191 |
| A40 | 39 | A91 | 90 | A142 | 141 | A193 | 192 |
| A41 | 40 | A92 | 91 | A143 | 142 | A194 | 193 |
| A42 | 41 | A93 | 92 | A144 | 143 | A195 | 194 |
| A43 | 42 | A94 | 93 | A145 | 144 | A196 | 195 |
| A44 | 43 | A95 | 94 | A146 | 145 | A197 | 196 |
| A45 | 44 | A96 | 95 | A147 | 146 | A198 | 197 |
| A46 | 45 | A97 | 96 | A148 | 147 | A199 | 198 |
| A47 | 46 | A98 | 97 | A149 | 148 | A200 | 199 |
| A48 | 47 | A99 | 98 | A150 | 149 | A201 | 200 |
| A49 | 48 | A100 | 99 | A151 | 150 |  |  |
| A50 | 49 | A101 | 100 | A152 | 151 |  |  |
| A51 | 50 | A102 | 101 | A153 | 152 |  |  |

**Question 20**

On a scale from 1 to 10, where 1=No impact at all, and 10=Very big impact, what is currently the **impact of your condition on your professional / working life?** Please tick.

| A1 | 1 | A6 | 6 |
| --- | --- | --- | --- |
| A2 | 2 | A7 | 7 |
| A3 | 3 | A8 | 8 |
| A4 | 4 | A9 | 9 |
| A5 | 5 | A10 | 10 |

**Question 21**

How has your condition impacted on your professional / working life?

| A1 | I miss many days of work | A10 | I feel isolated |
| --- | --- | --- | --- |
| A2 | I miss too many days of work and that resulted in not progressing / getting a promotion | A11 | I feel judged for taking days off work |
| A3 | My colleagues do not understand my condition or take it seriously | A12 | I cannot concentrate at work |
| A4 | I have been bullied at work | A13 | I worry that I lose my job |
| A5 | I did not get hired | A14 | I am not fully productive at work |
| A6 | I feel stigmatized | A15 | My condition does not impact my work performance |
| A7 | I have lost my job due to my condition | A16 | My condition does not impact my career |
| A8 | I have changed jobs because of my condition | A17 | None |
| A9 | I have changed my profession / career path because of my condition |  | |
